# A novel framework for designing a multi-DoF prosthetic wrist control using machine learning

**DOI:** 10.1038/s41598-021-94449-1

**Published:** 2021-07-22

**Authors:** Chinmay P. Swami, Nicholas Lenhard, Jiyeon Kang

**Affiliations:** 1 Department of Mechanical and Aerospace Engineering, University at Buffalo, Buffalo, NY 14260 USA; 2 Department of Computer Science and Engineering, University at Buffalo, Buffalo, NY 14260 USA; 3 Department of Biomedical Engineering, University at Buffalo, Buffalo, NY 14260 USA; 4 Department of Rehabilitation Science, University at Buffalo, Buffalo, NY 14214 USA

**Keywords:** Biomedical engineering, Electrical and electronic engineering, Mechanical engineering

## Abstract

Prosthetic arms can significantly increase the upper limb function of individuals with upper limb loss, however despite the development of various multi-DoF prosthetic arms the rate of prosthesis abandonment is still high. One of the major challenges is to design a multi-DoF controller that has high precision, robustness, and intuitiveness for daily use. The present study demonstrates a novel framework for developing a controller leveraging machine learning algorithms and movement synergies to implement natural control of a 2-DoF prosthetic wrist for activities of daily living (ADL). The data was collected during ADL tasks of ten individuals with a wrist brace emulating the absence of wrist function. Using this data, the neural network classifies the movement and then random forest regression computes the desired velocity of the prosthetic wrist. The models were trained/tested with ADLs where their robustness was tested using cross-validation and holdout data sets. The proposed framework demonstrated high accuracy (F-1 score of 99% for the classifier and Pearson’s correlation of 0.98 for the regression). Additionally, the interpretable nature of random forest regression was used to verify the targeted movement synergies. The present work provides a novel and effective framework to develop an intuitive control for multi-DoF prosthetic devices.

## Introduction

Myoelectric prostheses use a pair of surface electromyography sensors (EMG) to utilize electric motors for actuation of the terminal device. The most widely used transradial myoelectric prosthesis can only actuate power grasping and typically the wrist is fixed^1^. Limited mobility of these prosthetic devices causes compensatory trunk movement and unnatural upper body posture when using the terminal device to manipulate objects^2,3^. Furthermore, repeated excessive upper body movements generate early fatigue and pain which naturally leads to overuse of the intact limb^4,5^. Multiple survey studies indicate that the dissatisfaction factor of upper limb prostheses has been attributed to limited function, control strategy, and having higher weight^6–9^. Especially to increase the functionality of the prosthetic devices, state-of-the-art prosthetic designs have been developed to restore lost limb functions with multiple active degrees of freedom (DoF)^10–12^.

To actuate multi-DoF prosthetic devices, various control methods using EMG signals have been developed. One of the widely explored methods is the state machine approach which used two EMG signals to control single joint but allowed for switching between different joints by co-activation of both muscles^13^. However, these approaches lacked intuitive and simultaneous control of multiple DoF which hindered the dexterity of the hand movement during daily living tasks. To overcome the limitations of the state machine approach, pattern recognition based solutions used machine learning to identify the patterns present in the EMG signals generated during different motor tasks^13–15^. But due to the low degree of intuitiveness and increased cognitive burden^16^, its transition from the lab environment to daily use has been challenging. Furthermore, performance of EMG based controllers is limited due to electrode shift, variation in the force from the different pose, and transient changes in EMG due to muscle fatigue from long-term use^17^. These limitations have rekindled the search for alternative approaches to control multi-DoF prosthetic devices. Numerous studies have investigated alternative control strategies such as using ultrasound signals^18,19^, mapping the muscle deformation to the intended joint angle^20^, myokinetic control^21^, using the tongue as a joystick^22^, and movement based approaches^23^. Movement based control methods are shown to be intuitive by controlling the prosthesis using other joint movements instead of muscle signals^24^.

**Figure 1:**
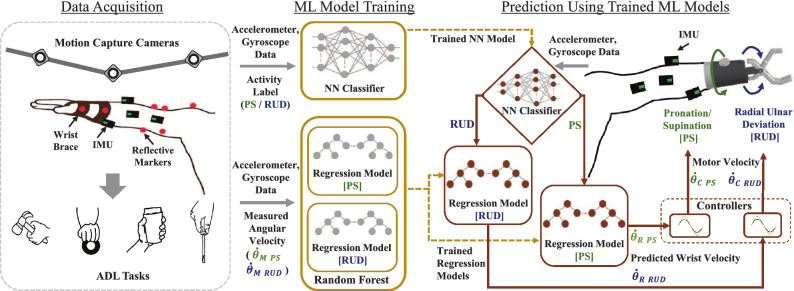
Overview of the proposed framework for modelling multi-DoF wrist control. Data collected from activities of daily living is used to train neural network classifier and regression models of radial/ulnar deviation (RUD) & pronation/supination (PS). Regression models and the control modules predict the angular velocity for controlling motors in a robotic prosthetic wrist. $\dot{\theta }_{M\_RUD}$ and $\dot{\theta }_{M\_PS}$ represent the measured angular velocities for RUD and PS computed using a motion capture system. $\dot{\theta }_{R\_RUD}$ and $\dot{\theta }_{R\_PS}$ represent angular velocities for RUD and PS predicted by regression models. $\dot{\theta }_{C\_RUD}$ and $\dot{\theta }_{C\_PS}$ are angular velocities generated by the controller modules for RUD and PS.

Movement based control approaches exploit the residual joint movements for the control of prosthetic joints using inertial measurement unit (IMU) sensors. Compensatory movement of the trunk and the arm was used to actuate prosthetic wrist pronation/supination to reduce these compensatory movements^25^. Other movement based control used existing movement synergies to control the distal joints using the proximal joints of the residual limb^24,26^. Using the movement synergies between the wrist and the shoulder, the controller allowed not only natural and intuitive control of prosthetic wrist pronation/supination but also reduced the cognitive burden on the prosthesis user^24^.

Especially, various regression models utilizing movement synergies between proximal and distal upper limb joints have been widely explored for the control of robotic prosthesis. Merad et al. modeled elbow flexion/extension by leveraging the natural co-ordination between the elbow and the shoulder^27^. Popovic et al.^23,28–30^ demonstrated the efficacy of controlling the wrist pronation/supination and elbow flexion/extension using shoulder movement. This was achieved by leveraging radial basis function networks (RBFN) to learn and map the movement synergies between the proximal and distal joints during reaching movements towards different target locations. Although the control approach was intuitive, manual switching between synergies for different targeted direction made the control inefficient. To avoid manual switching, multiple artificial neural networks were trained using all three DoF of the shoulder and scapuloclavicular movement^31^. These previous studies showed improved intuitive control, however, they had several limitations. First, they were limited to using the movement synergies for modeling only a single DoF of each upper limb joint. Employing movement synergies for modeling multi-DoF joint control has not been widely investigated yet. Second, the movement synergies were mostly modeled during simple reaching movements. However, tasks carried out during daily living do not comprise of only the reaching movements. Last, the previous regression based approaches were modelled based on the movement synergies from non-amputee participants. When this model was transferred to the amputee participant, the performance decreased because the residual limb movement of amputees is kinematically different from that of non-amputees^32^.

This study proposes solutions to overcome the limitations discussed above. First, we propose a novel framework that leverages multiple joint synergies for modeling control of a multi-DoF wrist and demonstrate its effectiveness. Second, the framework proposes to use the data obtained during ADL tasks to span different work spaces and improve the performance of the controller. Third, the inclusion of a wrist brace during the data collection is proposed to simulate the kinematics of amputees^3^. Fourth, the process of manually modeling complex joint synergies for multi-DoF control is eliminated as the framework incorporates machine learning. Furthermore, the interpretable nature of the machine learning algorithm enables quantifying the contribution of each component of the IMU sensors to the angular velocity of the wrist. Lastly, the proposed framework does not apply feature extraction on the IMU signals. This eliminates the time-consuming process of identifying the optimal features of the signals and minimizes the post-processing which typically leads to the integration of error from long-term use of the prosthetic arm and degrades the performance of the controller.

The core idea behind the proposed framework is to develop a control using machine learning which leverages the residual upper limb movement to allow for intuitive control of a multi-DoF prosthetic wrist. Therefore, movement synergies between the wrist and proximal joints are modeled using IMU sensors placed on the residual limb. For training the machine learning models, ADL tasks such as Drinking from a cup, Hammering a nail, Twisting screws, and Turning a pulley are used. These tasks are chosen because they are commonly used tasks in ADL performance measures such as Arm Motor Ability Test (AMAT), Activities Measure for Upper Limb Amputees (AM-ULA), and Southampton Hand Assessment Protocol (SHAP)^33–37^. To record the synergistic relationship, the wrist of the participants is braced during the data collection. This allowed to train the machine learning models using data acquired from the non-amputee participants emulating the absence of the active wrist movement. Due to its interpretable nature, random forest regression is used to map the residual limb movement to the intended velocity for radial/ulnar deviation and pronation/supination of the wrist. Rigorous off-line testing involving unknown participants’ data is conducted to assess the efficacy of the controller developed using the proposed framework. The residual upper limb movement will be mapped to the angular velocity of the respective wrist movement by the machine learning models. This angular velocity can be used as a control signal for a robotic prosthesis to actuate a multi-DoF wrist. Due to the use of the intuitive movement synergies for volitional control, we envision this novel framework for the development of the prosthetic controller, to contribute towards reduced compensatory movement and reduced mental burden on the prosthesis user. Furthermore, the framework can be easily extended to incorporate other additional upper limb movements including wrist flexion/extension.

## Results

The upper limb kinematics of ten non-amputee subjects was collected when they performed four activities of daily living (ADL). The ADL tasks required the subjects to perform wrist radial/ulnar deviation or pronation/supination. Motion capture data and IMU signals were used to train and test the machine learning models. Especially, for training the machine learning models the IMU signals were used without computing the joint angles or extracting any features from the signals.

As shown in Fig. 1, the presented framework for developing the controller consists of three main components: neural network classifier, random forest regression models, and control modules. The neural network classifier was trained to identify the intent for either radial/ulnar deviation (RUD) or pronation/supination (PS) based on the IMU signals. For training regression models, the measured angular velocities of RUD and PS were obtained using a motion capture system. Random forest regression models were trained with IMU signals as inputs and the measured angular velocity as outputs. Two regression models were individually trained to predict the angular velocities for RUD and PS movements respectively. Next, two control modules received the predicted velocity from the regression models for each wrist movement. The control modules compute angular velocities which will be used to control the velocity of the motors in a prosthetic arm. Each control module is comprised of an Algorithm 1 that augments the angular velocity to incite increased wrist movement. The control modules augment the wrist movement because the brace restrained the movement of the wrist. Since control modules can modulate the magnitude of predicted angular velocities, the importance of achieving low root mean squared error (RMSE) is reduced if the correlation coefficient is high^38^.

In the following sections, $\dot{\theta }_{M}$ will be used to represent the angular velocities observed using a motion capture system during ADL tasks (measured angular velocity) , $\dot{\theta }_{R}$ will be used to represent the angular velocity predicted by the regression models (predicted angular velocity), and $\dot{\theta }_{C}$ will be used to represent the angular velocity generated by the controller (controller output angular velocity).

**Figure 2:**
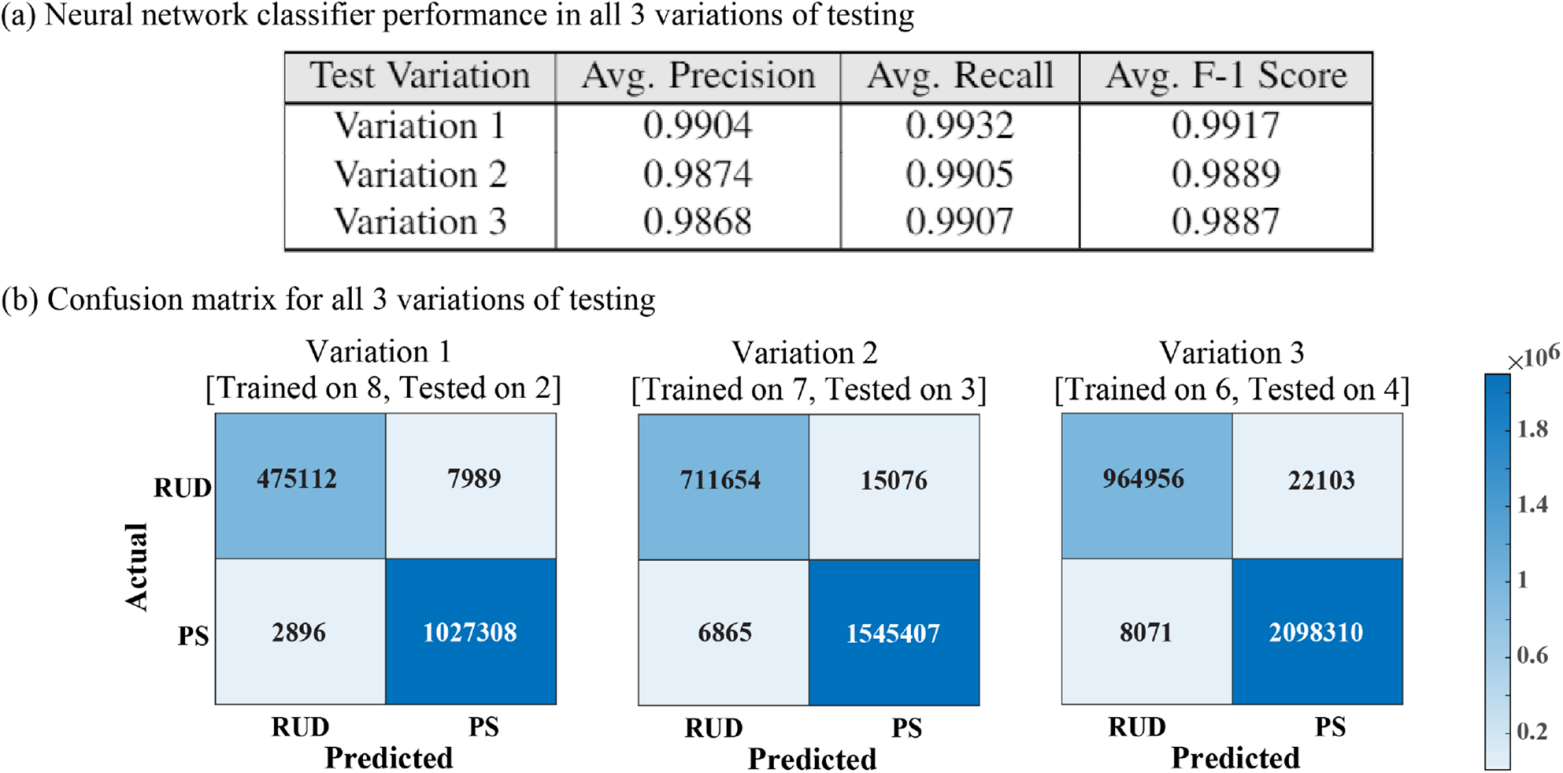
**(a)** Neural network classifier performance with precision, recall, and F-1 score averaged across all the test sets. Variation 1 involved testing on two unknown participants to the model, variation 2 involved testing on three unknown participants, and variation 3 involved testing on four unknown participants. **(b)** Confusion matrices depicting the classification performance of neural network models for all 3 variations of testing.

### Neural network classifier

The validation process of neural network classifier focused primarily on the ability of the model to accurately classify and generalize between subjects. Therefore, the model was trained using a set of randomly selected participants and tested on remaining participants that were unknown to the model. Figure 2a shows the observed classifier performance in the three variations of testing that were conducted. Each variation was repeated 30 times, each time generating a unique combination of participants. The performance of the model on each combination was averaged to indicate overall performance for the corresponding variation of testing. A detailed description of this process is discussed in the methods section. The performance of the model was evaluated using F-1 score which is the harmonic mean of precision and recall^39^. Precision evaluates the fraction of correctly classified instances among the ones classified as positive. The recall is a metric that quantifies the number of correct positive predictions made out of all positive predictions that could have been made. Figure 2a shows the precision, recall, and F-1 score averaged across all combinations for each variation of testing.

In the first variation of testing, an average F-1 score, precision, and recall of 0.99 were observed for different combinations of participants. Although a slight drop from 0.99 to 0.987 and 0.988 was observed in average precision and recall during the second variation of testing, the model’s performance was similar to the first variation of testing. Furthermore, the model’s performance in the third variation of testing was analogous to the second variation of testing even though it was trained with less number of participants. Between all the three variations of testing, the smallest F-1 score, precision, and recall of 0.95 were observed in the second variation for one of the combinations of participants. Further analysis of the model’s performance is shown in Fig. 2b representing the confusion matrix which indicates the number of correctly and incorrectly classified instances averaged across all combinations of the participants for each variation of testing.

**Figure 3:**
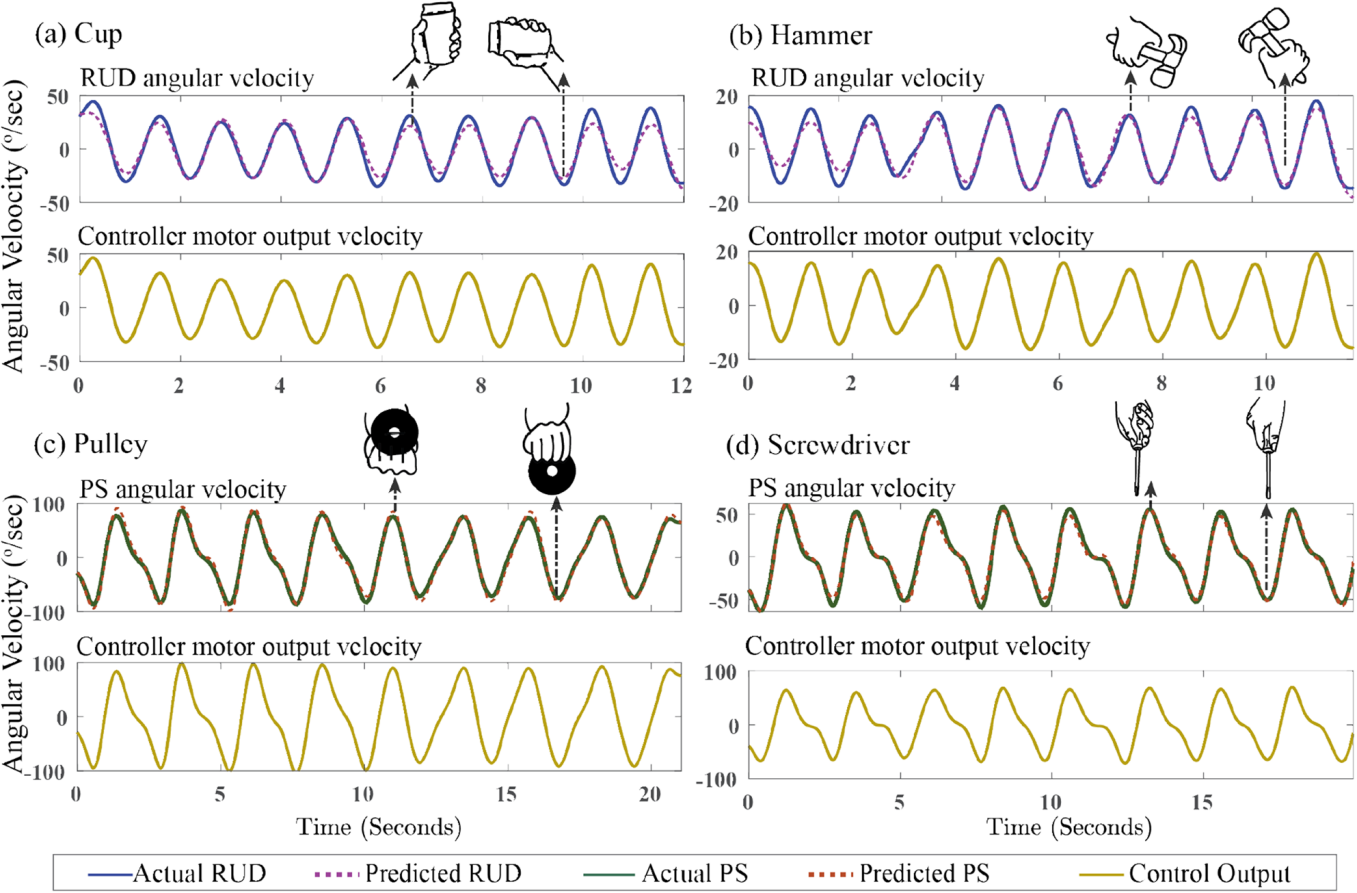
Measured Vs Predicted angular velocity and the controller generated angular velocity for one representative participant during each task. **(a)** Cup and **(b)** hummer tasks are designed for radial/ulnar deviation. **(c)** pulley and **(d)** screw driver tasks are designed for pronation/supination.

### Random forest regression model

The validation process of the regression models focused primarily on quantifying how far the regression model’s predictions were from the measured angular velocity. For assessing the robustness of the models, the models were not only trained/tested using the grouped data of all participants (generic model) but also trained/tested using each individuals’ data separately (individual model). Furthermore, to assess the model’s performance on unknown participants, the three variations of testing discussed in neural network section was also conducted. Fig. 3 illustrates the comparison between the measured angular velocity and the predicted angular velocity from the generic regression models for one representative participant during ADL tasks. Table 1a shows the performance of the regression models trained using all participants’ data and Table 1b shows the performance of the regression models in the three variations of testing.

In Table 1a, models trained using all participants’ data had an overall root mean squared error (RMSE) of 7.45 deg/s when averaged over all ADL tasks. The highest RMSE of 11.67 deg/s was observed for the pulley task. For radial/ulnar deviation related tasks RMSE of 4.84 deg/s was observed whereas pronation/supination related tasks, RMSE was 9.82 deg/s. Mean absolute error (MAE) between the peaks in measured angular velocity and the predicted angular velocity was also computed. The average peak MAE for all tasks was 2.34 deg/s with the highest MAE of 3.04 deg/s for the pulley task. Pearson’s correlation coefficient was also computed between the measured ($\dot{\theta }_M$) and the predicted ($\dot{\theta }_R$) angular velocity for each ADL task to evaluate the similarity in the patterns of the two signals. In Table 1a, the highest correlation of 0.99 was observed for the Pulley task whereas the lowest correlation of 0.97 was observed for the Hammer task. Lastly, the coefficient of determination for each trained tree was computed using 37% of the training data points that are unknown to the trees^40^ and was aggregated across the trees to indicate the performance of the random forest regression model, called as Out-of-Bag score^41^. An Out-of-Bag score of 0.99 was observed for both RUD and PS related tasks. As shown in Table 1b, when the models were tested on unknown participants in the three variations of testing, the RMSE values were in the range of 13.22 to 13.53 deg/s. Furthermore, Table 2 summarizes the RMSE values observed for the models that were trained and tested for data of each individual participant. The average RMSE of individual models was in the range of 4.4 to 12.4 deg/s, similar to the generic model trained with all participants’ data.

**Table 1:** (a) Performance measures for generic regression models trained with the data of all participants. (b) Performance measure for regression models trained with the data of a few participants and tested on unknown participants with three different variations.

(a) Performance of regression models on different ADLs			
Activity	RMSE	Peak MAE	Correlation
Cup	6.1067	2.1502	0.9894
Hammer	3.6077	1.6122	0.9714
Pulley	11.6656	3.0363	0.9902
Screwdriver	8.0218	2.4942	0.9810
Average	7.4464	2.3415	0.9833

(b) RMSE of regression models on unknown participants			
Activity	Variation 1	Variation 2	Variation 3
Cup	11.7418	11.8774	11.9929
Hammer	7.0046	7.0895	7.8180
Pulley	19.7644	20.5803	20.1278
Screwdriver	14.3792	14.1315	14.2167
Average	13.2225	13.4196	13.5388

Variation 1 tested on two unknown participant, variation 2 tested on three unknown participants, and variation 3 tested on four unknown participants. Units for RMSE and Peak MAE are deg/s.

**Table 2:** Table summarizing RMSE values between measured ($\dot{\theta }_{M}$) and predicted ($\dot{\theta }_{R}$) angular velocity of random forest regression models trained/tested using each individual participant’s data.

	Individual models RMSE (deg/sec)										
Activity	1	2	3	4	5	6	7	8	9	10	Avg.
Cup	6.252	3.984	4.874	5.924	11.618	3.179	7.836	5.215	10.234	7.795	6.691
Hammer	5.829	2.311	6.996	3.323	4.916	5.207	2.442	3.886	3.409	3.843	4.216
Pulley	21.905	8.158	10.769	5.412	12.550	14.321	15.765	15.201	13.253	7.417	12.475
Screwdriver	6.959	8.351	10.708	8.066	12.641	7.691	7.648	8.570	11.454	6.788	8.887

### Controller output

The brace limited the wrist movement and the controller utilizes the synergistic movement of the residual proximal joints to create wrist movement. To reduce the movement of proximal joints in the residual limb, the wrist movement needs to be amplified. Therefore, the control module was implemented, comprising of Algorithm 1, to augment the angular velocities predicted by the regression models. The validation process focused on assessing the similarity between the angular velocity measured by the motion capture system ($\dot{\theta }_{M}$) and the angular velocity generated by the controller ($\dot{\theta }_{C}$). Figure 3 depicts the angular velocity generated by the controller for one of the subjects during all four tasks.

An overall correlation of 0.9974 was observed between the measured and the controller generated angular velocity for all the ADL tasks. An average correlation of 0.9998 was observed for RUD and 0.9979 was observed for PS related tasks. These high correlation values indicate that the angular velocity generated by the controller closely follows the angular velocity measured by the motion capture system. But higher peaks were observed in the angular velocities generated by the controller when comparing with the magnitude of the measured angular velocities. This is expected because the angular velocities, predicted based on the movement synergies, are amplified by the controller with the intention of reducing the movement of the proximal joints in the residual limb.

## Discussion

The aim of this study is to demonstrate the feasibility of controlling multi-DoF wrist of a prosthetic arm by using the residual upper limb motion. A methodical off-line investigation was performed to present the robustness of the developed controller. A novel framework, illustrated by Fig. 1, is proposed where a neural network classifier and two random forest regression models were trained using IMU signals to model the movement synergies and predict the user’s intent and angular velocities for the wrist movement. The result of our study shows that the controller developed using the framework can be successfully used to generate the angular velocity for both RUD and PS, using only residual upper limb motion. Furthermore, the proposed framework has the advantage of not requiring feature extraction or joint angle computations. Since joint angles were not used to train the models, accumulation of errors due to integration was also minimized. Moreover, the models were designed with the data acquired from ADL tasks, instead of using simple reaching tasks as in previous studies^23,27–32^.

**Figure 4:**
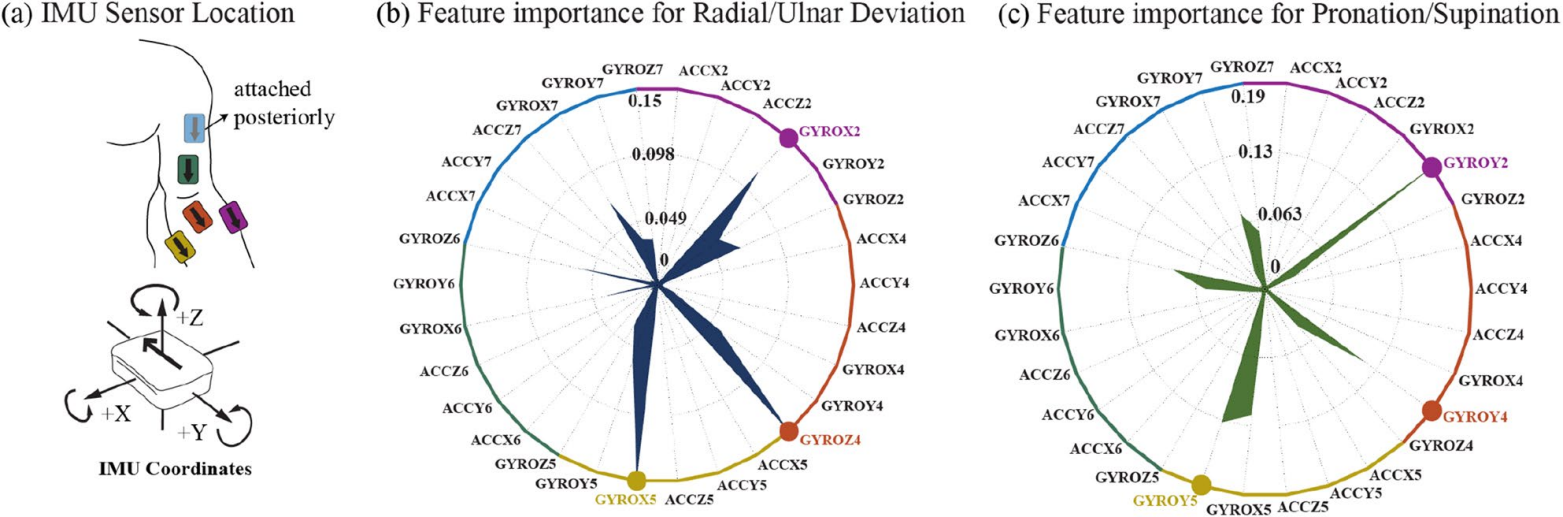
**(a)** IMU sensor locations on the upper limb. The faded sensor is attached on the posterior side of the upper limb. **(b)** Illustration of the important features contributing to predicting angular velocities for radial/ulnar deviation of the wrist. **(c)** Illustration of the important features for pronation/supination. The solid circles in the outer rim indicate features with highest contribution towards prediction of angular velocities.

**Figure 5:**
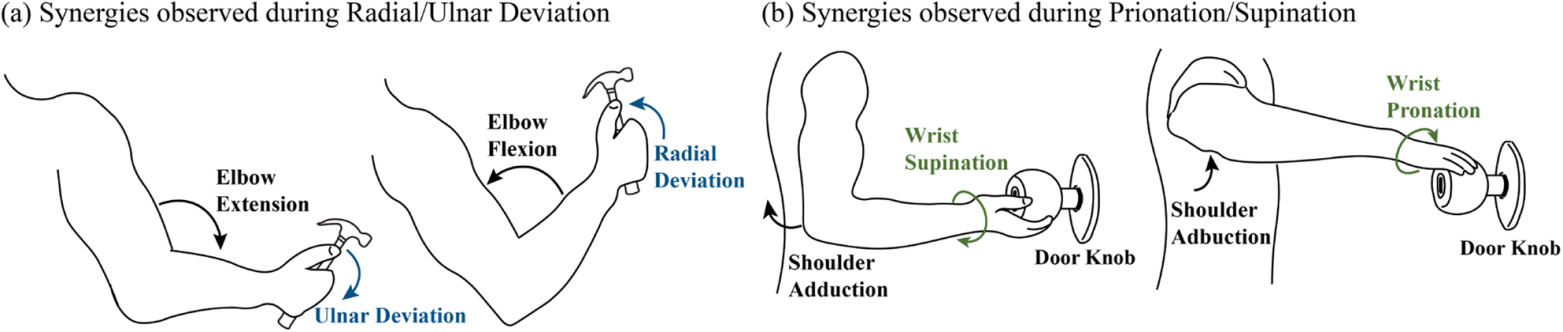
Postural synergies exhibited by the participants during hammering task and door knob (pulley in the experiment) rotating task.

The neural network classifier not only showed good classification performance but also generalized across unknown participants as shown in Fig. 2 indicating the F-1 score and confusion matrices. The neural network classifier, trained using only six participant’s data, was able to decode the intent of the 4 unknown participants to either perform radial/ulnar deviation or pronation/supination with a high accuracy of 98%. Augmenting the training data with more participant data only increased the classification accuracy as shown in Fig. 2a. This performance is comparable to the results reported in previous literature where finger and wrist posture were classified with the accuracy ranging from 80 to 99.99 % using various machine learning methods such as linear discriminant analysis (LDA), support vector machines (SVM) or artificial neural networks (ANN)^42,43^.

The random forest regression models were able to map the accelerometer and gyroscope signals to the angular velocity of RUD and PS with high precision as shown in Fig. 3 and Table 1a. Furthermore, the model’s success in exploiting the postural synergies and the robustness of the model was demonstrated by comparison of generic and individual model, in addition to the validation with hold out data sets. Among the four different ADLs, pulley task showed higher RMSE compared to other tasks. We believe that this observation is due to the need for complex movement involved in pulley task, spanning three dimensional workspace. However, a high correlation coefficient of 0.99 is observed for the Pulley task which indicates high degree of similarity between the measured and predicted angular velocities as shown in Table 1a. As control modules allow to change the magnitude of predicted angular velocities, high correlation coefficient is sufficient even though high RMSE values were observed^38^.

An overall correlation coefficient of 0.98 for random forest regression and 0.99 for the control modules was observed in our study. Kaliki et al. reported a correlation coefficient of 0.97 by using shoulder movement as an input to a neural network for predicting wrist pronation/supination angles during reaching tasks^31^. Merad  et al. modeled the postural synergies when the participants performed reaching movements using radial basis function network to control elbow flexion/extension which resulted in a correlation coefficient of 0.88. Another study using time delayed neural network and EMG signals observed a correlation coefficient of 0.68 between the measured and predicted joint angles for wrist pronation/supination^44^. Other previous studies that used EMG signals and regression methods for wrist flexion/extension and radial/ulnar deviation reported correlation coefficients in the range of 0.70 to 0.98^45–49^. We believe that using common synergistic patterns present in different tasks and individuals improved the prediction capability of our random forest regression models^50^.

For designing a prosthetic controller with multiple DoFs, identification of multiple movement synergies and manually mapping the movement synergies for each DoF are required which can be challenging. The proposed framework allows identifying the synergistic relationship between the residual and prosthetic joints by using the data collected from different ADLs. The random forest regression algorithm was adopted to identify features that contributed to the prediction. In Fig. 4b,c, the important features identified by random forest regression models were used to validate the movement synergies. The features identified as important by the random forest regression were in harmonious to the previously published movement synergies^3,26,51^. As shown in Fig. 5, radial/ulnar deviation related ADL tasks involved elbow flexion/extension. For pronation/supination related ADL tasks, shoulder ab/adduction instigated the axial rotation in forearm. Figure 4b,c demonstrate the framework’s capability to successfully learn these movement synergies to predict the angular velocities for multi-DoF wrist movements. Additionally, the information about the sensor contribution can be used to determine the optimal number of sensors for the prosthetic controller with high DoF or assist in troubleshooting the controller in case the model’s behaviour is unexpected. Lastly, as the angular velocity and the acceleration measured by the IMU sensors contribute to the predicted angular velocity of the wrist, the controller is less sensitive to different upper arm postures.

Although the proposed framework was shown to yield high level of off-line performance, the study was limited to off-line evaluation. Therefore, future studies will be focused on empirical analysis in a real-time virtual environment and assess the reliability of the controller. Real-time testing will be also conducted to garner in-depth user experience of operating the suggested controller. We will also explore long-term use of our controller and validate the performance when it is repeatedly used. This study did not analyze the effects of discarding less significant features on the performance of regression and classification models. Further studies are required for analyzing the effects of removing such features on the performance of models and how the explainable nature of the models can be used in reducing the number of sensors.

Using this study as a starting point, we plan to further analyze the efficacy of the framework by recruiting both amputee and non-amputee participants. A comparison between the models developed on non-amputee participants and the models developed on the amputee participants using the suggested framework would provide more insight into the efficacy of the framework. This comparison will test our hypothesis that developing machine learning models using movement synergy data, acquired by emulating amputee participants with a brace, has better prediction capabilities and higher chances of success with amputees compared to the models developed on data acquired without using the brace. Furthermore, we plan on extending the proposed framework for modeling 3-DoF active wrist movements by including flexion/extension. Lastly, if the framework is found to be successful in amputee trials, we plan to implement the developed controller in a robotic prosthesis and conduct experiments with amputee participants to assess the performance of the framework using standard clinical measures.

## Conclusion

In this paper, a novel framework for the control of multi-DoF prosthetic wrist was presented. The framework uses movement synergies present in the upper limbs by leveraging a neural network classifier and random forest regression to allow multi-DoF control of a prosthetic wrist. This movement synergy based actuation of the prosthetic limb has a higher chance of creating natural wrist movements during ADL tasks and reduce compensatory movement. The model was trained using raw IMU data without any post-processing such as converting IMU data in global coordinates to compute joint angles or extracting features. Furthermore, the random forest algorithm allows for easy inclusion of sensor data with different physical metrics due to its robust structure. In the present study, the models’ performance on the dataset recorded during trials involving four different ADL tasks showed high performance. Classification accuracy of 99% was achieved by the neural network classifier. The random forest regression models had an RMSE of 7.44 deg/s with a correlation coefficient of 0.98 between the measured and predicted angular velocity. The observed results are quite promising, propounding the use of the proposed framework in developing the controller for multi-DoF prosthetic wrist. The proposed framework can be easily extended to additional upper limb movements. Future work will involve real-time testing of the proposed framework using a virtual reality environment and amputee participants.

## Methods

### Human experiment set-up

The experimental protocol was approved by the Institutional Review Board (IRB) of University at Buffalo. All participants were over 18 years old. They were informed about the research procedures and signed a written consent form approved by the IRB before participating in the study. All experiments were performed in accordance with relevant guidelines and regulations. Ten healthy subjects (three females and seven males with an average weight of 70.20 ± 17.31 kg and an average height of 173.29 ± 12.07 cm) performed tasks that were designed to emulate activities of daily living (ADL). An off-the-shelf brace was modified to restrict the wrist motion so that the movements made by the proximal joints will be emphasized. A motion capture system with ten infrared cameras (Vicon, UK) sampling at 100 Hz was used to record the upper limb movements. In addition, five Trigno Avanti IMU sensors (Delsys, US) sampling at 148 Hz were used to acquire movement data.

**Figure 6:**
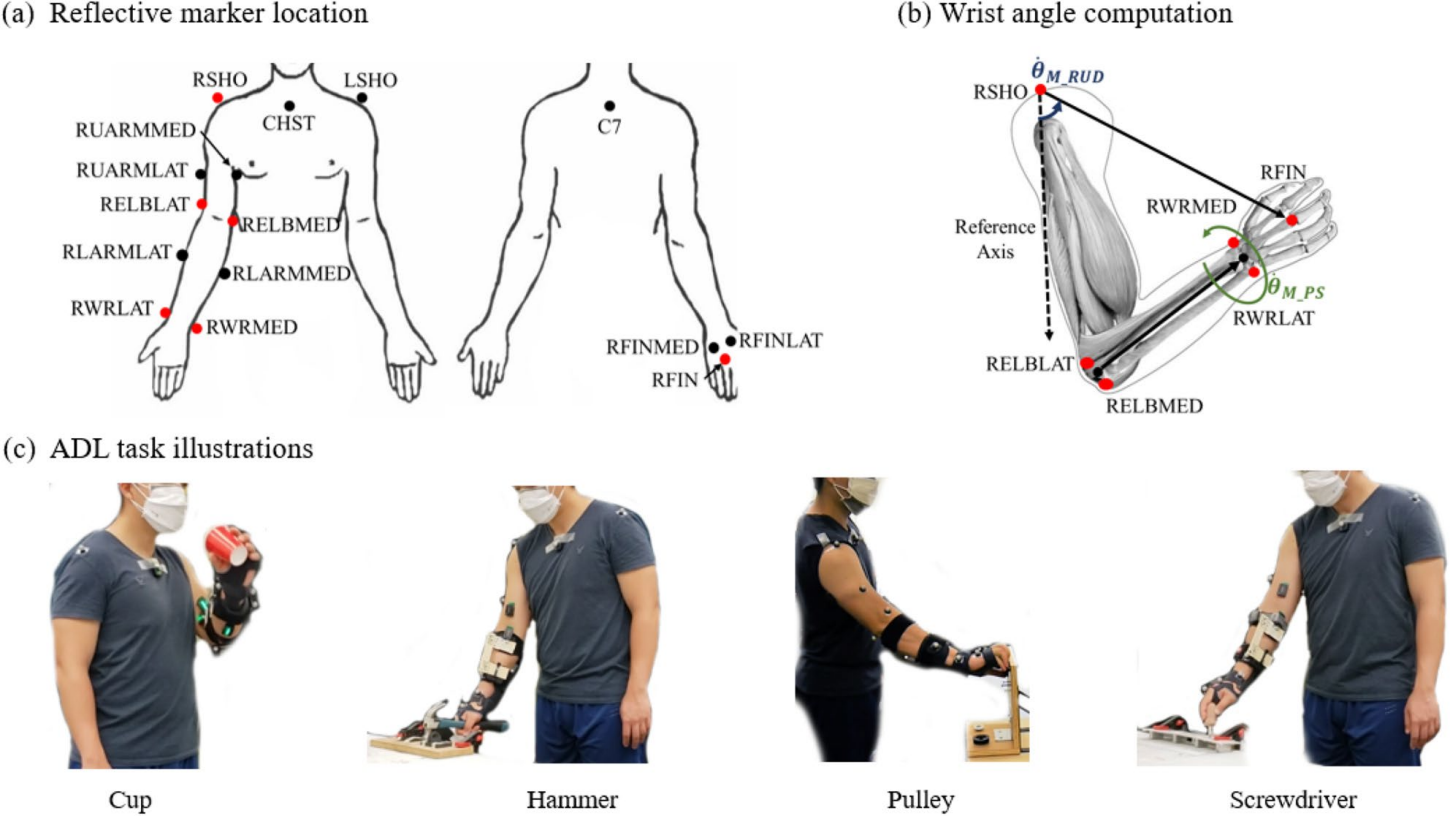
**(a)** Location of reflective markers attached on the upper limb during the experiment. **(b)** Illustration of basis vectors used for computation of angular velocity for radial/ulnar deviation and pronation/supination of the wrist. **(c)** A participant performing four different ADL tasks with the wrist brace.

### Experimental procedure

Fifteen reflective markers were attached on bony landmarks as shown in Fig. 6a. Five IMU sensors were placed on the participants as shown in Fig. 4a. Y-axes of IMU’s were aligned with the long bones of the arm and the Z-axes of IMU’s were perpendicular to the skin. The ADL tasks were primarily designed to acquire data pertaining to two wrist motions: radial/ulnar deviation and pronation/supination^52,53^. Drinking from a cup and hammering a nail were designed for acquiring wrist radial/ulnar deviation data. Twisting the screws and turning the pulley were designed to acquire pronation/supination data. For each ADL task, five trials were conducted, and each trial started and ended with participants maintaining a default pose known as a T-Pose where participants stand with both shoulders abducted at 90$^{\circ }$, elbows completely extended, and palms facing down. Furthermore, for the tasks involving clockwise and anti-clockwise movement, participants transitioned to T-Pose for a brief period after performing the clockwise movement and before initiating anti-clockwise movement. A metronome was used to have uniform speed throughout the trials. The experiment took an average of 2.5 h to complete. Following were the four ADL tasks for each trial:

- Drinking from a cup: This task was adopted from the AM-ULA performance assessment measure where participants mimicked the action of drinking from a cup^34^. The participants had to pick up the cup placed on the table, bring it closer to the mouth and then try to tilt the cup to simulate drinking. The participants mimicked the drinking action for 10 times.
- Hammering a nail: This task is similar to ‘Use a hammer and nail’ task from the AM-ULA performance assessment measure and involved the participant hitting a nail with a hammer at a constant speed^34^. The participant picked up the hammer and positioned the hammer on top of the nail mounted on a wooden board. The participants hit the nail ten times.
- Twisting screws: This task was designed to simulate ‘Rotate a Screw’ task from SHAP performance assessment protocol^36^, and consisted of twisting 3 screws in the clockwise and anti-clockwise directions in the transverse plane. Participants picked up the screwdriver and first twisted each screw three times in a clockwise direction, and then twisted the three screws in an anti-clockwise direction.
- Turning a pulley: This activity was designed to simulate turning a doorknob or a bulb which are activities performed on a daily basis. These tasks are a part of multiple performance assessment measures^34, 36, 54^. This task consisted of turning a pulley in the clockwise and anti-clockwise directions in the frontal plane. Participants reached the pulley and turned the pulley five times in a clockwise direction and then turned the pulley five times in an anti-clockwise direction.

### Data pre-processing

For computing angle representing radial/ulnar deviation, two vectors, one from RSHO to LSHO and the other from RSHO to C7, were used to form a plane. A vector perpendicular to this plane was used as the reference axis and had the origin at RSHO. Another vector from RSHO to RFIN was created and the angle between the reference axis and this vector was used as the angle representing the radial/ulnar deviation. For pronation/supination of the wrist, joint angles of the distal segment (forearm) relative to the proximal segment (upper arm) were computed. The coordinate system described by the ISB standards was used to define the coordinates for the forearm segment^55^. For the forearm, the Y-axis was defined by the vector from wrist midpoint to the elbow midpoint. The Z-axis was a vector from wrist midpoint to RWRLAT and the X-axis was the vector perpendicular to the plane formed by the Z and Y-axis. For the proximal segment, the Y-axis was defined by a vector from the elbow midpoint to the RSHO marker. The Z-axis was a vector from elbow midpoint to RELBLAT and the X-axis was the vector perpendicular to the plane formed by the Z and Y-axis. Using the Z-X-Y Euler rotation sequence, the rotation of the forearm segment around Y-Axis was used as angle for wrist pronation/supination. Figure 6b illustrates the detailed coordinate axes used for computing both wrist angles.

The kinematic data captured by the motion capture system was filtered using 4th order low-pass Butterworth filter with a cutoff frequency of 6 Hz. The radial/ulnar deviation and pronation/supination angles were filtered with a low-pass Butterworth filter having a cutoff frequency of 1 Hz. Angular velocities were numerically computed and then passed through the same low-pass filter. The signals generated by the IMU sensors were filtered using a 3rd order low-pass Butterworth filter with a cutoff frequency of 1 Hz.

### ML model training

Machine learning was employed to achieve two things. First, the intent of the user for either radial/ulnar deviation or pronation/supination will be identified. Second, machine learning model will predict the angular velocity required to provide the desired motor speed for the respective wrist movement. To achieve this, first, a neural network based binary classifier was developed to initially classify the user’s intent to either deviate radius/ulna or pronate/supinate the wrist. Then two random forest regression models were trained to predict the angular velocity, one for radial/ulnar deviation and the other for pronation/supination. Figure 1 illustrates the training process along with the inputs to the ML models and their outputs. Systematic off-line evaluation was conducted on the trained machine learning models by excluding some participants’ data from the training set.

#### Classification model

A densely connected neural network with two hidden layers was created using Python 3.7 and Keras 2.4.3^56^ to accomplish the task of binary classification. The input to the neural network classifier was IMU signals from the sensors shown in Fig. 4a and the output was probabilities for each class. The class with the highest probability was considered as the user’s intent. The first hidden layer consisted of ten neurons and the second hidden layer consisted of five neurons. The hidden layers used ReLU (rectified linear unit)^57^ as the activation function and the output layer used Softmax as the activation function^58^. Binary cross entropy was used as a loss function with Adam (adaptive moment estimation) as the optimizer^59^. The number of hidden layers and neurons was determined by trial and error to avoid overfitting of the model. Neural networks trained using smaller batch sizes have been shown to generalize well^60^. Therefore, through trial and error, a small batch size of eight was used for training the classifier.

To validate the efficacy of the model, three variations of testing were performed. In the first variation of testing, 30 unique combinations of eight participants were generated by random selection. For each of the 30 combinations, eight participants’ data was used to train the model and the two excluded participants’ data was used to assess the quality of the fit. For the second variation of testing, a procedure similar to the first variation was adopted however, instead of eight participants, seven unique participants were used to form 30 unique combinations. And in the third variation, a similar procedure but with six participants was performed.

Macro-Averaged F-1 score, Macro-Averaged precision, and Macro-Averaged recall along with confusion matrix provided quantitative measures indicating the performance of the trained classifier models across different variations. F-1 score is the harmonic mean of precision and recall which indicates the accuracy with which the classifier identifies a class and is robust to the class imbalance in dataset^61^. Precision evaluates the fraction of correctly classified instances among the ones classified as positive. The recall is a metric that quantifies the number of correct positive predictions made out of all positive predictions that could have been made. A detailed description of these assessment methods can be found in^39^.

#### Regression model

When evaluated against a variety of supervised learning algorithms using different performance criteria and data sets, random forest performed better than most of the other popular learning algorithms^62^. They have been fairly successful in inferring from different bio signals^63^ and have been used in modeling controllers for prostheses^64–66^.

Random forest is a type of ensemble learning algorithm based on the ‘divide and conquer’ strategy and consists of two core components: CART (classification and regression trees) split criterion and Bagging^41^. CART split criterion regulates the construction of each individual tree in the forest. Bagging is a method in which bootstrapped samples are generated from the original data set and each sample is used to fit a different tree in the forest. The random forest algorithm is comprised of three main steps. First, for a given training set *D*, *T* sets of *n* elements are sampled from D with replacement. Second, for each subsample, a decision tree is constructed using CART. In random forest, CART is modified to have a fixed number of randomly selected features for splitting the data. The number of randomly selected features used for splitting the subsample is held constant throughout the process. The quality of the split is assessed using mean squared error (MSE), and the set of randomly selected features that yields the best split is selected. Furthermore, the trees are not pruned and are allowed to grow to their largest possible extent or to some predefined threshold. Lastly, prediction *p* for a new input *r* is computed by aggregating the outputs of the trained regression trees $R_1,R_2 ... R_T$ in the forest as indicated by Eq. (1)^67^.

1 $$\begin{aligned} p(r) = \frac{1}{T}\sum _{t=1}^{T} R_t(r) \end{aligned}$$

Another advantage of random forest is computation of feature importance which helps in identifying features that have a strong influence in predicting the angular velocity, $\dot{\theta }_R$. The feature importance is measured by the overall decrease in variance when split on a feature averaged across all the trees. Thus, weighted decrease in variance corresponding to split along the feature $f_j$ is computed and is averaged over all trees, where $f_j$ is the *j*th feature in dataset *D*. A detailed description of the algorithm can be found in^41,68^. The feature importance was used to identify the signals of the IMU sensors which influenced the most in predicting the angular velocity $\dot{\theta }_R$. Furthermore, it also allowed us to validate the movement synergies by checking if the sensors identified as important were harmonious to the observed movement synergies.

The random forest regression models were developed using Python 3.7 and Scikit-learn^69^. Using GridsearchCV from Scikit-learn, it was found that a forest with 50 trees having a maximum depth of 40 performed well in predicting the angular velocities for both radial/ulnar deviation and pronation/supination. Two training data sets were created, one for radial/ulnar deviation and the other for pronation/supination containing IMU data collected during ADLs. To assess the model’s predictions, three different approaches were used. The first approach involved using 4 trials of all the participants to train the model and using the 5th trial to test it. In the Second approach, models were trained and tested for each individual instead of one generic model trained using all participants’ data. The third approach was similar to the three variations of testing discussed in the neural network classifier section where the regression models were trained on a few participants and tested on the remaining unknown participants. The model’s performance was judged by measuring the similarity between measured ($\dot{\theta }_M$) and the predicted ($\dot{\theta }_R$) angular velocity for the wrist using root mean squared error (RMSE) and mean absolute difference between the peaks. Pearson’s correlation coefficient *R* was also computed because it is independent of the unit, enabling comparison with previous studies^70^. Furthermore, due to bagging when the individual trees are trained, around 37% of the training data points are unknown to the trees in the model^40^. Hence, these unknown data points are used to assess the performance of the trained tree and aggregated across the trees to indicate the performance of the entire model also known as Out-of-Bag score^41^. Performance on Out-of-Bag samples was also tracked by computing the coefficient of determination, R${^2}$.

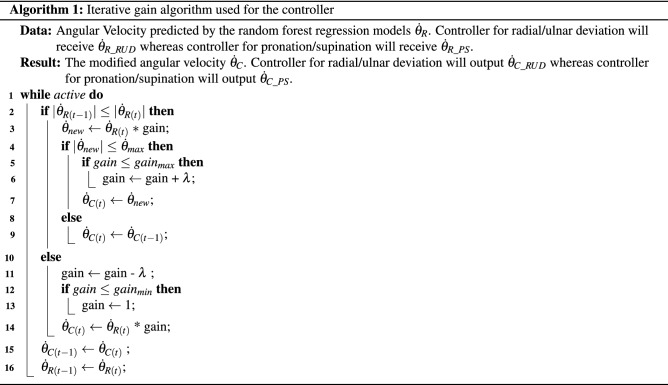



### Control module design and testing

As shown in Fig. 1, two control modules were developed for each radial/ulnar deviation and pronation/supination of the wrist, in order to increase the wrist movement and reduce the required range of motion of residual limb to control the wrist. Each control module receives the predicted angular velocity ($\dot{\theta }_{R}$) from the regression models. The control module is comprised of an algorithm that augments the predicted angular velocity ($\dot{\theta }_{R}$) by a given value *gain* to increase the wrist movement. Now instead of keeping a constant value for *gain*, we increase or decrease the *gain* by $\lambda$ at time *t* by comparing the predicted angular velocity ($\dot{\theta }_{R}$) at time *t* and $t-1$. The *gain* value had upper ($gain_{max}$) and lower ($gain_{min}$) bounds to ensure that controller generates angular velocity ($\dot{\theta }_{C}$) that isn’t too large or too low. From trial and error, $\lambda =0.00008$, $gain_{max}=2$ and $gain_{min}=1$ were used to compute the desired motor velocity in the controller algorithm. Furthermore, the maximum angular velocity is controlled by $\dot{\theta }_{max}$. Algorithm 1 depicts the pseudo-code of the iterative gain algorithm.

## Data Availability

The data from IMU sensors and motion capture system during four different ADLs will be available upon request to the corresponding author (jiyeonk@buffalo.edu).
